# Repetitive transcranial magnetic stimulation for improving function after stroke

**DOI:** 10.1590/1516-3180.20131316T2

**Published:** 2013-12-01

**Authors:** Zilong Hao, Deren Wang, Yan Zeng, Ming Liu

## Abstract

**BACKGROUND:**

It had been assumed that suppressing the undamaged contralesional motor cortex by repetitive low-frequency transcranial magnetic stimulation (rTMS) or increasing the excitability of the damaged hemisphere cortex by high-frequency rTMS will promote function recovery after stroke.

**OBJECTIVE:**

To assess the efficacy and safety of rTMS for improving function in people with stroke.

**METHODS:**

*Search methods:* We searched the Cochrane Stroke Group Trials Register (April 2012), the Cochrane Central Register of Controlled Trials (CENTRAL) (The Cochrane Library 2012, Issue 4), the Chinese Stroke Trials Register (April 2012), MEDLINE (1950 to May 2012), EMBASE (1980 to May 2012), Science Citation Index (1981 to April 2012), Conference Proceedings Citation Index-Science (1990 to April 2012), CINAHL (1982 to May 2012), AMED (1985 to May 2012), PEDro (April 2012), REHABDATA (April 2012) and CIRRIE Database of International Rehabilitation Research (April 2012). In addition, we searched five Chinese databases, ongoing trials registers and relevant reference lists.

*Selection criteria:* We included randomized controlled trials comparing rTMS therapy with sham therapy or no therapy. We excluded trials that reported only laboratory parameters.

*Data collection and analysis:* Two review authors independently selected trials, assessed trial quality and extracted the data. We resolved disagreements by discussion.

**MAIN RESULTS:**

We included 19 trials involving a total of 588 participants in this review. Two heterogenous trials with a total of 183 participants showed that rTMS treatment was not associated with a significant increase in the Barthel Index score (mean difference (MD) 15.92, 95% CI -2.11 to 33.95). Four trials with a total of 73 participants were not found to have a statistically significant effect on motor function (standardized mean difference (SMD) 0.51, 95% CI -0.99 to 2.01). Subgroup analyses of different stimulation frequencies or duration of illness also showed no significant difference. Few mild adverse events were observed in the rTMS groups, with the most common events being transient or mild headaches (2.4%, 8/327) and local discomfort at the site of the stimulation.

**AUTHORS' CONCLUSIONS:**

Current evidence does not support the routine use of rTMS for the treatment of stroke. Further trials with larger sample sizes are needed to determine a suitable rTMS protocol and the long-term functional outcome.

**This is the abstract of a Cochrane Review published in the Cochrane Database of Systematic Reviews (CDSR) 2013, issue 5, Art. No. CD008862. DOI:** 10.1002/14651858.CD008862.pub2 (http://cochrane.bvsalud.org/cochrane/main.php?lib=COC&searchExp=Repetitive%20and%20transcranial%20and%20magnetic%20and%20stimulation%20and%20for%20and%20improving%20and%20function%20and%20after%20and%20stroke&lang=pt). 

**The abstract (English, Portuguese and French languages) is available from:** http://onlinelibrary.wiley.com/doi/10.1002/14651858.CD008862.pub2/abstract.
